# Ultrasound-Assisted Extraction: Unlocking the Antibacterial Potential of *Coptis chinensis* Franch. Against ESBL-Producing *Enterobacterales*

**DOI:** 10.3390/molecules30224331

**Published:** 2025-11-07

**Authors:** Ching Ching Hui, Fred Wang Fat Lee, Wesley Chin Ho Lung, Kai Chung Fan, Ivan Tak Fai Wong, Gilman Kit Hang Siu, Yeuk Lung Chow, Ping Lung Chan, Siu-Mui Ng, Ling Shi, Sai Wang Seto, Franklin Wang Ngai Chow, Emily Wan Ting Tam

**Affiliations:** 1 School of Science and Technology, Hong Kong Metropolitan University, Ho Man Tin, Kowloon, Hong Kong SAR, China; 2 Department of Health Technology and Informatics, The Hong Kong Polytechnic University, Kowloon, Hong Kong SAR, China; 3 School of Nursing and Health Sciences, Hong Kong Metropolitan University, Ho Man Tin, Kowloon, Hong Kong SAR, China; 4 School of Biomedical Sciences, The University of Western Australia, Perth, WA 6009, Australia; 5 NICM Health Research Institute, Western Sydney University, Penrith, NSW 2751, Australia; 6 Research Centre for Chinese Medicine Innovation, The Hong Kong Polytechnic University, Hong Kong SAR, China

**Keywords:** ultrasound-assisted extraction, ESBL-producing *Enterobacterales*, *Coptis chinensis* Franch., berberine, antimicrobial resistance

## Abstract

The global rise of multidrug-resistant Extended Spectrum β-Lactamase Producing-*Enterobacterales* (ESBL-PE) poses a critical threat to public health, driving the urgent need for alternative therapeutic approaches. This study evaluates the antimicrobial properties of 20 Chinese medicinal herbs against 14 ESBL-PE strains from seven bacterial species, utilizing three extraction methods: traditional water decoction, 80% ethanol maceration, and 50% ethanol with ultrasound-assisted extraction (UAE). Among the herbs tested, *Coptis chinensis* Franch. demonstrated the most potent anti-ESBL-PE activity, effectively inhibiting multiple strains, including *Escherichia coli*, *Klebsiella pneumoniae*, *Hafnia alvei*, *Citrobacter freundii* and *Proteus hauseri*. *C. chinensis* extracts obtained via UAE exhibited superior antibacterial activity to the other two extraction methods, attributed to enhanced extraction efficiency and improved bioactive compound yields. Specifically, UAE increased the extraction yield of alkaloids in *C. chinensis* by 80.9%, compared to the ethanol maceration method, and the increase in berberine, the key antimicrobial compound, was 75.4%. Berberine demonstrated significant antibacterial effects against ESBL-PE strains, while other phytochemicals in *C. chinensis* extracts have an additional effect with berberine, further amplifying the overall antimicrobial activity. These findings highlight that the UAE is a promising method for enhancing the therapeutic potential of *C. chinensis* and other Chinese medicinal herbs against multidrug-resistant bacteria.

## 1. Introduction

*Enterobacterales*, an order of Gram-negative bacteria, includes several well-known pathogens such as *Salmonella*, *Klebsiella*, *Escherichia coli*, *Enterobacter*, and *Shigella* [1]. These genera predominantly inhabit the intestinal tract of humans and animals, and some members are also found in the environment [2]. Extended-Spectrum β-Lactamase-Producing-*Enterobacterales* (ESBL-PE) produces an enzyme known as extended-spectrum beta-lactamase (ESBL) that degrades commonly used antibiotics, rendering them ineffective for treating infections [3]. The widespread use of third- and fourth-generation cephalosporins has driven the emergence of ESBL-PE [4]. The global spread of ESBL-PE has escalated significantly, posing a critical threat to public health. ESBL enzymes can be easily transmitted among *Enterobacterales* members via plasmid-mediated gene transfer. This mechanism also facilitates the spread of resistance to other commonly used antibiotics, such as aminoglycosides, fluoroquinolones, and sulphonamides, contributing to the rise of multidrug-resistant ESBL-PE [5,6]. Consequently, patients often require last-resort antibiotics, such as carbapenems, to treat infections caused by multidrug-resistant bacteria [7,8]. However, the increased use of carbapenem has led to a rapid rise in carbapenem-resistant *Enterobacterales* [9]. This alarming trend underscores the urgent need for effective strategies to prevent the emergence of multidrug-resistant bacteria and to discover novel anti-ESBL-PE agents.

The development of new antimicrobial drugs is a complex, time-intensive, and expensive process. It often requires more than 7 years to secure regulatory approval, and the clinical approval rate is less than 20% [10]. Because of this slow process and the urgent need for an effective method to prevent the emergence of multidrug-resistant bacteria, scientists have increasingly turned to Chinese medicinal herbs as a promising alternative. These herbs and their active ingredients have demonstrated multiple antibacterial and bactericidal properties [11,12,13,14]. They are effective against antibiotic-resistant bacteria, including methicillin-resistant *Staphylococcus aureus* (MRSA) [15,16,17,18]. The antibacterial and bactericidal properties of Chinese medicinal herbs are primarily achieved through mechanisms such as inhibiting biofilm formation in drug-resistant bacteria, suppressing the efflux pump system, eliminating resistant plasmids, and altering bacterial permeability [19]. For instance, extracts from *Reynoutria japonica* Houtt. have been shown to damage the cell walls and cytoplasmic membranes of nosocomial drug-resistant bacteria such as *Staphylococcus aureus*, *Pseudomonas aeruginosa* and *Acinetobacter baumannii* [13]. Similarly, extracts from *Sophora moorcroftiana* (Benth.) Baker exhibit antibacterial properties by suppressing the efflux pump system of drug-resistant *Staphylococcus aureus* [20]. These findings suggest that Chinese medicinal herbs could serve as viable alternatives or adjuncts to conventional antibiotics. Compared to conventional antibiotics, Chinese medicinal herbs offer several advantages, including more abundant resources, easier availability, reduced likelihood of drug resistance, and a higher diversity of active ingredients [21]. Additionally, they exhibit fewer side effects and target a broader range of mechanisms [22].

The main active ingredients in traditional Chinese herbs that exhibited antimicrobial properties include flavonoids, alkaloids, phenols, quinones, and tannins. These phytochemicals have demonstrated synergistic effects with various antibiotics in treating drug-resistant bacterial infections [21]. The type and quantity of bioactive compounds isolated from Chinese medicinal herbs depend on the extraction methods, affecting their antibacterial efficacy and antimicrobial potency. Various forms of herbal extracts have been developed over the long history of Chinese medicinal herbs. Among these, decoction in water and maceration in alcohol are the two most common methods due to their simple application, easy preparation, low cost, and proven medicinal efficacy [23,24]. More recently, ultrasound-assisted extraction (UAE) has emerged as a powerful technique for enhancing the extraction of bioactive compounds due to its superior efficiency and extraction capacity [25,26,27,28]. UAE offers several advantages, including shorter extraction times, reduced solvent usage, lower energy consumption, operation at low temperatures, increased cost-effectiveness, and higher environmental sustainability. While UAE has been shown to significantly enhance the antioxidant activity of extracts from various medicinal herbs compared to traditional methods such as water decoction and alcohol maceration [29,30], its impact on the antibacterial properties of herb extracts remains underexplored. The efficacy and types of bioactive compounds extracted by UAE may also vary among different Chinese medicinal herbs. This study aims to address these gaps by investigating the application of UAE in extracting bioactive compounds from Chinese medicinal herbs with potential antibacterial activity against multidrug-resistant bacteria. Unlike previous research, which primarily focuses on UAE’s role in extracting biomolecules for anti-cancer or antioxidant purposes, this work emphasizes its potential in combating antimicrobial resistance. We hypothesize that UAE may extract a greater quantity of bioactive compounds from Chinese medicinal herbs, resulting in higher antibacterial efficacy compared to conventional extraction methods, though the results may vary depending on the specific herb. This study will compare the efficacy of various extraction methods, identify the most potent herbal extracts, and determine their primary active anti-ESBL-PE compounds. The work will provide scientific information for the development of Chinese medicinal herbs as potential therapeutic agents to combat multidrug-resistant bacteria.

## 2. Results

### 2.1. The Antibiotic-Resistant Profile of ESBL-PE Strains

This study included 14 multidrug-resistant ESBL-PE strains (B1–B14) isolated from food samples, comprising three *E. coli* (B1–B3), four *Klebsiella pneumoniae* (B4–B7), two *Hafnia alvei* (B8–B9), two *Citrobacter freundii* (B10–B11), one *Citrobacter braakii* (B12), one *Proteus hauseri* (B13), and one *Enterobacter cloacae* (B14) (Table 1). Species identification was confirmed using Matrix-Assisted Laser Desorption Ionization Time-Of-Flight Mass Spectrometry (MALDI-TOF MS), with all strains achieving a score of ≥2.0, indicating successful and reliable identification. Antimicrobial susceptibility testing using a panel of 16 antibiotics revealed that all strains were multidrug-resistant, exhibiting resistance to three or more antibiotic classes. ESBL production was confirmed using the double disc synergy test and further validated by whole-genome sequencing, which identified at least one beta-lactamase resistance gene in each isolate (Table 2). Notably, 9 out of the 14 ESBL-PE strains harbored multiple beta-lactamase resistance genes. All beta-lactamase resistant genes identified in these strains belonged to Ambler classes A, C, or D [31]. Among these, only one class D beta-lactamase resistance gene, *blaOXA*, was detected in *K. pneumoniae* (B6). All other beta-lactamase resistance genes belonged to classes A or C. Furthermore, all ESBL strains were resistant to ampicillin and cefotaxime but remained susceptible to meropenem and doripenem (Table 2). Multiple efflux pump genes were also detected in 10 strains, including all strains of *E. coli*, *K. pneumoniae*, *C. freundii,* and *E. cloacae*. The presence of these efflux pump genes suggests that efflux-mediated mechanisms may contribute to the multidrug resistance observed in these strains. In contrast, no efflux pump genes were identified in *H. alvei*, *C. braakii,* or *P. hauseri* strains. Their multidrug resistance profiles may instead be attributed to other mechanisms.

### 2.2. UAE Extracts of Coptis chinensis Franch. Exhibited the Most Potent Anti-ESBL-PE Activity

#### 2.2.1. Bacterial Growth Inhibition

Disc diffusion results revealed that 6 out of 20 tested Chinese medicinal herbs, namely *Lithospermum erythrorhizon* Siebold & Zucc., *Sarcandra glabra* (Thunb.) Nakai, *Scutellaria baicalensis* Georgi, *C. chinensis* Franch., *Prunella vulgaris* L., and *Magnolia officinalis* Rehder & E.H.Wilson, demonstrated broad-spectrum anti-ESBL-PE activity across all three extraction methods. However, their efficacy varied depending on the herbs and the extraction method used (Table 3). The remaining 14 herbs showed no bacterial growth inhibition effects. Among the extraction methods, the water decoction extracts of *L. erythrorhizon*, *S. glabra,* and *M. officinalis* exhibited the highest antibacterial activity, in terms of both the inhibition zone diameter (IZD) and the number of susceptible ESBL-PE strains. Specifically, these water decoction extracts showed antibacterial effects against seven, six, and five ESBL-PE strains, respectively. Conversely, the ethanol with UAE extracts of *C. chinensis* and *P. vulgaris* outperformed the water decoction and ethanol maceration extracts, displaying the highest antibacterial activity, affecting eight and six ESBL-PE strains, respectively. For *S. baicalensis*, both the water decoction and ethanol with UAE extracts exhibited comparable antimicrobial activity, each inhibiting six ESBL-PE strains. These findings indicate that there is no universal extraction method that consistently enhances the antibacterial activity of all herbal extracts, and the effectiveness is herb-specific. However, when comparing the two ethanol-based methods (ethanol maceration and ethanol with UAE), the UAE method consistently demonstrated higher antibacterial activity across all six herbs, as measured by both IZD and the number of susceptible ESBL-PE strains. Notably, among all the herb extracts, the UAE extract of *C. chinensis* exhibited the strongest anti-ESBL-PE activity, with an IZD ranging from 6.95 ± 0.10 mm to 13.38 ± 0.22 mm against eight ESBL-PE strains, followed by *L. erythrorhizon*, with an IZD ranging from 7.29 ± 0.04 mm to 9.49 ± 0.09 mm against seven ESBL-PE strains. Importantly, *C. chinensis* was the only herb that demonstrated antimicrobial activity against ESBL-producing *E. coli* (Table 3).

#### 2.2.2. Determination of Minimum Inhibitory Concentration (MIC) of Herb Extracts

To further evaluate the anti-ESBL-PE activities of the six Chinese medicinal herbs *L. erythrorhizon*, *S. glabra*, *M. officinalis*, *C. chinensis*, *P. vulgaris* and *S. baicalensis* that demonstrated antibacterial effects in the disc diffusion assay, the MIC values of all their extracts were determined against ESBL-PE strains. Six strains, randomly selected from species that showed susceptibility in the disc diffusion assay, included *E. coli* (B2), *K. pneumoniae* (B6), *H. alvei* (B8), *C. freundii* (B10), *C. braakii* (B12), and *P. hauseri* (B13). The MIC results revealed that all herb extracts exhibited antibacterial effects against the six selected ESBL-PE strains, with MIC values ranging from 0.78125 to 25 mg/mL. However, the ethanol maceration extract of *S. glabra* did not show any anti-ESBL-PE activity against the *E. coli* B2 strain within the tested concentration range (Table 4). When comparing the two ethanol-based extraction methods (ethanol maceration and UAE), the UAE method generally yielded lower MIC values, aligning with the disc diffusion assay results and indicating that UAE is a more effective extraction method than traditional maceration for most herbs. However, for *S. baicalensis*, the MIC values obtained using UAE were similar to those from ethanol maceration, indicating that the difference between the two methods for *S. glabra* is minimal. When comparing the antibacterial effects of water decoction and UAE extracts from the same herb, the MIC values against all ESBL-PE species were either identical or differed by one-fold. Among the six herbs, *C. chinensis* exhibited the most potent antimicrobial effect against ESBL-producing *E. coli*, with MIC values of 1.5625 mg/mL for the UAE extract and 3.125 mg/mL for the water decoction extract. In contrast, the other five herbs (*L. erythrorhizon*, *S. glabra*, *S. baicalensis*, *P. vulgaris*, and *M. officinalis*) showed significantly higher MIC values (12.5–25 mg/mL) (Table 4). Furthermore, the UAE extract of *C. chinensis* exhibited the strongest overall inhibitory effect on ESBL-PE strains, with MIC values ranging from 1.5625 to 3.125 mg/mL. The finding indicates that *C. chinensis* has the highest potential for development as an anti-ESBL-PE agent. Interestingly, some herb extracts that did not exhibit antibacterial effects against certain ESBL strains in the disc diffusion assay (Table 3) showed activity in the MIC test (Table 4). This discrepancy suggests the disc diffusion assay has certain limitations, such as diffusion constraints, which can affect the detection of antibacterial activity.

#### 2.2.3. UAE Extracts of *C. chinensis* Contained the Highest Alkaloid Content

**Figure 1:**
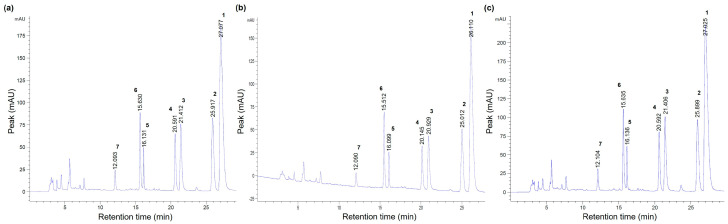
The chromatographic peaks of seven alkaloid compounds from *Coptis chinensis* Franch. extracted by (**a**) water decoction; (**b**) 80% ethanol maceration; and (**c**) 50% ethanol with ultrasound assisted extraction. (Peaks 1–7 are berberine, palmatine, coptisine, epiberberine, jatrorrhizine, columbamine, and groenlandicine, respectively).

**Figure 2:**
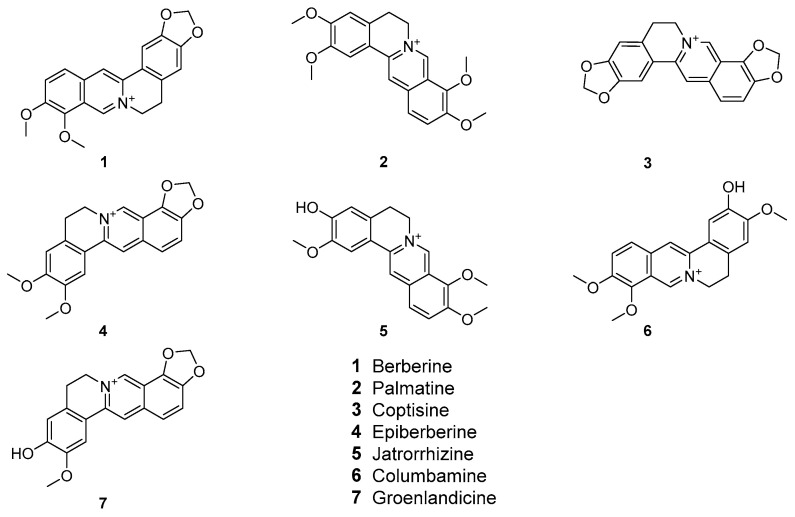
The molecular structures of the seven most abundant alkaloids identified from *Coptis chinensis* Franch. Extracts.

Since *C. chinensis* displayed the most potent antimicrobial activities among all 20 herb extracts, the differences in phytochemical compositions of *C. chinensis* extracted by different methods were investigated using HPLC-UV analysis (Figure 1). The results revealed that the seven most abundant alkaloids in the three *C. chinensis* extracts were berberine, epiberberine, palmatine, coptisine, jatrorrhizine, columbamine, and groenlandicine, listed in descending order of abundance (Figure 2 and Table 5). These alkaloids constituted 42.4%, 29.9% and 54.1% of the total content in the water decoction, ethanol maceration and UAE extracts, respectively. Among these alkaloids, berberine was the most abundant compound across all *C. chinensis* extracts, accounting for 17.9%, 13.4%, and 23.5% of the total content in the water decoction, ethanol maceration, and UAE extracts, respectively (Table 5). Notably, the UAE method consistently extracted higher levels of each of the seven alkaloids compared to the other two methods, explaining why the UAE extracts of *C. chinensis* exhibited the strongest antimicrobial activity (Table 4). When comparing alkaloid extraction yields between the ethanol maceration method and the UAE methods, UAE was found to significantly enhance the extraction yield of bioactive alkaloids in *C. chinensis* by 80.9% compared to the ethanol maceration method. Specifically, the extraction yield of berberine was increased by 75.4%, highlighting the efficiency of UAE in maximizing the recovery of key antimicrobial compounds. The antimicrobial efficacy of *C. chinensis* might closely correlate with the abundance of alkaloid content in the extracts. Specifically, the higher concentration of alkaloids, particularly berberine, in the UAE extracts may contribute to their superior antibacterial effects against ESBL-PE strains.

#### 2.2.4. The Antibacterial Activity of *C. chinensis* Is Predominantly Attributed to Berberine, with Additional Contributions from Other Phytochemicals

To investigate whether berberine is the primary antibacterial compound in the *C. chinensis* UAE extracts against ESBL-PE strains, the MIC values of berberine alone were compared to those of the *C. chinensis* extracts. Table 6 showed that the MIC value of berberine alone was 0.75 mg/mL, which was lower than the MIC value of the *C. chinensis* extracts against ESBL-producing *E. coli* (1.5625 mg/mL). However, when the equivalent concentration of berberine in the *C. chinensis* extract was calculated, the MIC value of the equivalent berberine in the *C. chinensis* extracts was 0.368 mg/mL, which was significantly lower than that of berberine alone. Similar results were observed for other ESBL-PE strains, including *K. pneumoniae*, *H. alvei*, *C. freundii*, *C. braakii*, and *P. hauseri* (Table 6). These findings suggest that, while berberine is the primary antibacterial agent in *C. chinensis*, other phytochemicals present in the extracts exert a synergistic or additional effect on berberine, significantly enhancing the overall antibacterial activity against ESBL-PE strains.

## 3. Discussion

The global rise of multidrug-resistant ESBL-PE has become a critical public health concern, necessitating the exploration of alternative therapeutic strategies. This study identified 14 multidrug-resistant ESBL-PE strains from food samples, each confirmed as multidrug-resistant and exhibiting resistance to multiple antibiotic classes, including key cephalosporins such as ampicillin and cefotaxime (Table 1). These findings reinforce evidence that food can serve as a significant reservoir for antibiotic resistance genes with zoonotic and clinical implications [32,33,34]. In addition to ESBL production, most isolates carried efflux pump genes, indicating multifactorial resistance mechanisms that involve both enzymatic degradation and active efflux. The coexistence of these pathways likely facilitates survival against diverse antibiotics and may complicate treatment options for ESBL-PE infections. Given this resistance landscape, the exploration of alternative antimicrobial agents is timely and necessary.

Traditional Chinese medicinal herbs, with well-documented pharmacological activities, have garnered particular interest due to their rich and diverse phytochemical content, including flavonoids, tannins, alkaloids, phenolics, sulfur compounds, and terpenoids [22,35,36,37,38,39,40]. However, the efficacy of these herbs depends on both the quality and quantity of bioactive compounds, which in turn are affected by the types of herbs and extraction methodologies. Traditional decoction, which involves boiling herbs, is supported by substantial clinical evidence for its efficacy [41]. This method is particularly effective at extracting water-soluble compounds, such as saponins, flavonoids and polyphenols, which are key bioactive components with antimicrobial properties [42,43]. Additionally, the boiling process can promote the formation of new compounds with synergistic effects [41]. However, prolonged exposure to high temperatures may degrade volatile and heat-sensitive molecules. Maceration, another widely used method for preparing herbal medicines, involves soaking herbal material in ethanol at an appropriate concentration at room temperature for an extended period. This process softens and breaks down plant cell walls, facilitating the release of soluble bioactive metabolites. The absence of heat helps preserve thermolabile and volatile compounds [44]. Ethanol maceration is particularly effective at extracting hydrophilic compounds such as alkaloids, flavonol, tannins, and terpenoids, many of which exhibit antimicrobial properties [45,46]. However, this method has limitations, including lengthy extraction times and relatively low extraction efficacy. UAE has emerged as a superior alternative for extracting active compounds from medicinal plants. UAE enhances solvent penetration into plant cells and improves mass transfer, accelerating the release of bioactive compounds [28,47].

In this study, several Chinese medicinal herbs, including *L. erythrorhizon*, *S. glabra*, *M. officinalis*, *C. chinensis*, *P. vulgaris,* and *S. baicalensis*, exhibited broad-spectrum anti-ESBL-PE activity across all extraction methods, as demonstrated by disc diffusion tests and MIC assays. The antimicrobial compounds in these herbs probably derive from distinct phytochemical classes. The naphthoquinone shikonin in *L. erythrorhizon*, terpenoids and organic acids in *S. glabra*, potent flavonoids in *S. baicalensis*, alkaloids (especially berberine) in *C. chinensis*, triterpenoids in *P. vulgaris*, and the lignan honokiol in *M. officinalis*, have demonstrated antibacterial and wound-healing properties in previous studies [48,49,50,51,52,53,54,55]. Notably, among the extraction methods, UAE consistently exhibited superior antibacterial activity compared to ethanol maceration across all six herbs. The enhanced efficiency of UAE is likely due to its ability to accelerate the disruption of plant cell walls, thereby facilitating the release of bioactive compounds [47]. This method significantly increased the extraction yield of bioactive compounds across diverse phytochemical groups, including naphthoquinones, terpenoids, flavonoids, alkaloids, triterpenoids, and lignans, all of which are known for their antimicrobial properties. HPLC-UV analysis further validated that UAE significantly increases the extraction yield of bioactive alkaloids in *C. chinensis* by 80.9% compared to the traditional ethanol maceration method. Specifically, the extraction yield of berberine was increased by 75.4%, highlighting the effectiveness of UAE in maximizing the recovery of key antimicrobial compounds.

Among the herbs tested, *C. chinensis* emerged as the most potent against ESBL-PE, demonstrating the broadest antibacterial effects, as evidenced by IZD values, MIC values, and the number of ESBL-PE strains inhibited. The UAE extract of *C. chinensis* effectively inhibited various ESBL-PE strains, including *E. coli*, *K. pneumoniae*, *H. alvei*, *C. freundii*, and *P. hauseri*. Berberine, an isoquinoline alkaloid identified in *C. chinensis*, is a key active ingredient with well-documented antibacterial, antiviral, and anti-inflammatory properties [56]. It has been traditionally used to treat intestinal infections such as acute gastroenteritis, cholera, and bacillary dysentery [57,58]. Interestingly, the study revealed that while berberine alone exhibits antimicrobial activity, the whole *C. chinensis* extract demonstrated even greater efficacy, indicating potential synergistic or additive interactions among co-extracted phytochemicals. This synergy likely enhances cell membrane permeability, disrupts efflux mechanisms, or interferes with other bacterial resistance pathways. Further investigation is needed to identify these synergistic phytochemicals, which could aid in the development of anti-ESBL-PE agents capable of combating multidrug-resistant bacteria.

This study provides valuable insight into the relationship between extraction methodology and antimicrobial efficacy, suggesting that UAE is a promising technique to maximize the therapeutic potential of Chinese medicinal herbs, particularly *C. chinensis*, against multidrug-resistant ESBL-PE. These findings support the inclusion of UAE-extracted *C. chinensis* as a candidate for further development. Future investigations should focus on optimizing UAE parameters, characterizing synergistic phytochemicals within *C. chinensis*, and validating efficacy through in vivo trials. Additionally, investigating the molecular pathways targeted by these alkaloids, such as their effects on bacterial cell walls, efflux pump systems, biofilm formation, and plasmid-mediated resistance, will be crucial. Mechanistic studies using transcriptomics could provide deeper insights into how these compounds disrupt bacterial resistance mechanisms. Overall, the findings highlight the potential of UAE to advance natural alternatives to traditional antibiotics, presenting a promising avenue for addressing the escalating challenge of ESBL-PE infections.

## 4. Materials and Methods

### 4.1. ESBL-PE Strains

Multidrug-resistant ESBL-PE strains were isolated from food samples, including pork, chicken, beef, sashimi, and vegetable, purchased from the Hong Kong market in 2022. All food samples were stored at 4 °C immediately after purchase and processed on the same day. A 25 g portion of each sample was homogenized in a stomacher (Seward stomacher 400, Worthing, UK) with a 1:10 dilution of phosphate-buffered saline (PBS). The homogenized samples were plated onto MacConkey Agar (MCA) and Violet Red Bile Glucose Agar (VRBGA) (Merck, Darmstadt, Germany). Plates were incubated aerobically at 37 °C for 24 h. Bacterial colonies from plates showing growth were sub-cultured onto nutrient agar plates and incubated at 37 °C for 24 h.

The identification of *Enterbacterales* strains was carried out by using MALDI-TOF MS with the MALDI Biotyper Sirius system (Bruker, Billerica, MA, USA), following the methodology described in our previous publication [59]. Briefly, a bacterial colony was mixed with 1 µL of matrix solution and allowed to dry on a stainless-steel target plate. Identification was then conducted using MALDI-TOF MS, and the resulting spectra were analyzed and compared against the Bruker Biotyper database using the MBT Compass Library (version 12.0.0.0). The presence of ESBLs was initially screened through antimicrobial susceptibility testing and subsequently confirmed by molecular profiling. Antimicrobial susceptibility testing was conducted using a panel of antibiotics, including ampicillin, cefepime, cefotaxime, cefoxitin, ceftazidime, ceftriaxone, chloramphenicol, ciprofloxacin, colistin, gentamicin, imipenem, meropenem, nalidixic acid, tetracycline, co-trimoxazole, and doripenem (Sigma-Aldrich, St. Louis, MO, USA). The ESBL phenotype was confirmed using the double-disc synergy test in accordance with the CLSI guidelines [60]. Isolates exhibiting resistance to three or more antibiotic classes were classified as multidrug-resistant strains [61]. To further characterize the strains, whole-genome sequencing was performed to identify ESBL-resistant genes and efflux pump genes. Genomic DNA was extracted using the QIAamp BiOstic Bacteremia DNA Kit (Qiagen, Hilden, Germany), and DNA library preparation was conducted following the SQK-RBK110.96 protocol (Oxford Nanopore Technologies, Oxford, UK). The pooled barcoded library was purified using AMPure XP beads (Beckman Coulter Inc., Brea, CA, USA). After adaptor ligation, the library pool was loaded onto R9.4.1 flow cells and sequenced on a Nanopore GridION sequencer (Oxford Nanopore Technologies) for 48 h using the super-accurate (SUP) base-calling mode. A minimum sequencing depth of 50× coverage was achieved, and high-quality sequence reads were retained after filtering. Genome assembly was performed using Dragonflye (v1.0.13), and antimicrobial resistance genes, including efflux pump genes, were identified using AMRFinderPlus (v3.10.30). Fourteen multidrug-resistant ESBL-PE strains (Table 6) were finally selected as representative *Enterobacterales* for this study based on their prevalence in the community, clinical significance, and multidrug-resistant profiles.

### 4.2. Chinese Medicinal Herbs

Twenty different types of Chinese medicinal herbs were selected based on their traditional antibacterial pharmacological functions and historical usage for evaluation of their anti-ESBL-PE properties (Table 7). The herbs were sourced from Tsang Fook Kee Medicine Company Limited, a retail shop in Hong Kong, with their origins traced to various cities across China. The authenticity of the herbs was verified by Chinese Medicinal Pharmacist Dr. Yeuk-Lung Chow.

### 4.3. Extraction of Chinese Medicinal Herbs

The dried herbs were coarsely ground using a blender, and each herb was subjected to three different extraction methods: water decoction, 80% ethanol maceration, and 50% ethanol extraction with UAE [63,64]. For the decoction extraction, 25 g of ground herbs were heated with 250 mL of distilled water for 25 min at 100 °C with stirring. The decocted solution was filtered immediately after cooling to room temperature. For the 80% ethanol maceration, 25 g of ground herbs were mixed with 250 mL of 80% ethanol and agitated for 72 h. The maceration process was repeated once, and the extracts from both rounds were combined. For the UAE method, 25 g of ground herbs were mixed with 250 mL of 50% ethanol and subjected to ultrasonic irradiation at 60 °C for 50 min with 100 W power in an ultrasonic bath (Bransonic M3800H-E, Brookfield, CT, USA). All extracts were filtered through a 0.45 μm microporous filter membrane. After filtration, the extracts were evaporated under vacuum at 50 °C using a rotary evaporator. The extracts were re-suspended in deionized water, freeze-dried and stored in an electronic desiccator until further experimentation. Freeze-drying was essential to quantify the concentration required for performing the disc diffusion test and MIC test.

### 4.4. Determination of Inhibition Zone of Herb Extracts by Disc Diffusion Methodology

Each herb extract was evaluated for anti-ESBL-PE activity against the 14 different ESBL-PE strains using the disc diffusion method, with reference to CLSI guidelines [60]. Briefly, bacterial culture was adjusted to a 0.5 McFarland standard and evenly spread across Mueller–Hinton agar (MHA) plates using sterile swabs. Herb extracts were dissolved in Mueller–Hinton broth (MHB) containing 5% DMSO_(aq)_ and filtered through a 0.22 µm syringe filter. A 6 mm blank disc was soaked with 20 µL of herb extract, resulting in a final concentration of 8 mg of herb extract per disc and placed on the MHA surface. Each test plate comprised eight discs, including one positive control, one negative control, and six discs treated with herb extracts. The positive control was a commercial gentamicin 10 µg antibiotic disc, while the negative control was a disc containing 5% DMSO_(aq)_. Plates were incubated at 37 °C for 18–22 h. The antibacterial activity of the Chinese medicinal herbs was assessed by measuring the inhibition zone diameter (IZD) around the discs using a caliper. The assay was performed in triplicate.

### 4.5. Determination of the Minimum Inhibitory Concentration (MIC) of Herb Extracts and Berberine

The MICs of Chinese medicinal herb extracts and berberine (ChemFaces, Wuhan, China) against ESBL-PE strains were determined following the methodology described by Chassagne et al. (2019), in accordance with EUCAST and CLSI standards [60,63,65]. MIC testing was conducted using 96-well plates, with the final concentration of DMSO_(aq)_ in each well kept below 5%. Herb extracts and berberine were tested at concentrations ranging from 0.39 to 25 mg/mL. The initial bacterial concentration in each test well, except for the negative control, was standardized to 1.5 × 10^6^ CFU/mL. Negative control wells contained herb extract or berberine without bacterial inoculation, while positive control wells contained bacterial inoculation without herb extract or berberine. The plates were incubated at 37 °C for 18 h. MIC values were determined visually by assessing the lowest concentration of herb extract or berberine that inhibited visible bacterial growth. All tests were conducted in triplicate.

### 4.6. Detection and Quantification of Alkaloid Compounds in C. chinensis Extracts Using High-Performance Liquid Chromatography-Ultraviolet (HPLC-UV) Spectrophotometry

HPLC-UV analysis of *C. chinensis* extracts was performed based on a previously published method with minor modifications [66]. For each extract, 0.1 g of *C. chinensis* was mixed with 25 mL of a hydrochloric acid and methanol solution (1:100 *v*/*v*). The total weight of the mixture was recorded, and the sample was ultrasonicated for 30 min. After cooling, the mixture was reweighed, and any weight loss was replenished with the same hydrochloric acid and methanol solution. The solution was then filtered through a 0.45 μm syringe filter, and the filtrate was transferred into a 2 mL vial for chromatographic analysis.

Chromatographic analysis was performed using an Agilent 1290 Infinity UPLC System equipped with a diode array detector (DAD) (Agilent Technologies, Santa Clara, CA, USA) and an XBridge C18 chromatographic column (4.6 × 150 mm, 5 μm) (Waters, Wexford, Ireland). The column temperature was maintained at 25 °C. The mobile phase consisted of acetonitrile (A) and 0.03 mol/L ammonium acetate solution containing 0.1% triethylamine and 0.6% ammonium hydroxide (%*v*/*v*) (B). The gradient program was set as follows: 0–16 min, 10–22% A; 16–30 min, 22–24% A; 30–33 min, 24–100% A. The injection volume for each sample was 2 µL. The flow rate was set at 0.5 mL/min for the first 15 min, then increased to 1.0 mL/min. The detection wavelength was set at 270 nm. The detected phytochemical compounds were validated using different reference standards. All the solvents used in HPLC-UV detection were of analytical grade and were purchased from Sigma-Aldrich, USA. The reference standards of berberine, palmatine, coptisine, epiberberine, columbamine, jatrorrhizine, and groenlandicine were purchased from ChemFaces, China. Following chromatographic analysis, the yields of alkaloids in the CR extract were calculated using the following formula:

$$\frac{Weight\;of\;alkaloids\;in\;C.\;chinensis\;extract\;(mg)\;}{Total\;weight\;of\;C.\;chinensis\;extract\;(mg)}\times 100\%$$

To compare the alkaloid extraction yields between the ethanol maceration method and the UAE method, the following formula was applied:

$$\frac{\%\;total\;alkaloids\;content\;in\;UAE\;extract\;-\;\%\;total\;alkaloids\;content\;in\;maceration\;extract\;\;}{\%\;total\;alkaloids\;content\;in\;maceration\;extract}\times 100\%$$

## Figures and Tables

**Table 1:** The fourteen ESBL-PE strains used in this study.

Isolate	Species	Source	Date of Collection	Food Origin
B1	*Escherichia coli*	Pork	August 2022	China
B2	*Escherichia coli*	Chicken	April 2022	Thailand
B3	*Escherichia coli*	Beef	August 2022	China
B4	*Klebsiella pneumoniae*	Beef	August 2022	China
B5	*Klebsiella pneumoniae*	Pork	May 2022	China
B6	*Klebsiella pneumoniae*	Beef	June 2022	China
B7	*Klebsiella pneumoniae*	Beef	July 2022	China
B8	*Hafnia alvei*	Beef	August 2022	USA
B9	*Hafnia alvei*	Pork	June 2022	Spain
B10	*Citrobacter freundii*	Sashimi	May 2022	Norway
B11	*Citrobacter freundii*	Vegetable	June 2022	Japan
B12	*Citrobacter braakii*	Sashimi	August 2022	Vietnam
B13	*Proteus hauseri*	Chicken	May 2022	China
B14	*Enterobacter cloacae*	Vegetable	June 2022	Japan

**Table 2:** Antibiogram of ESBL-PE strains.

Isolate	Species	Beta-Lactamase Resistant Gene	Efflux Gene	Antibiogram															
				AMP	FEP	CTX	FOX	CAZ	CRO	C	CIP	CS	CN	IPM	MEM	NA	TE	SXT	DOR
B1	*E. coli*	*bla*CTX-M-14, *bla*EC, *bla*SHV, *bla*TEM	*acrF*, *emrD*, *mdtM*, *silA*	R	SDD	R	S	R	R	R	R	I	R	S	S	S	R	R	S
B2	*E. coli*	*bla*EC, *bla*CTX-M-1	*acrF*, *emrD*, *mdtM*	R	R	R	S	S	R	S	S	R	S	S	S	S	R	S	S
B3	*E. coli*	*bla*CTX-M-14, *bla*EC, *bla*TEM	*acrF*, *emrD*, *mdtM*, *silA*	R	SDD	R	S	S	R	R	I	I	S	S	S	I	R	R	S
B4	*K. pneumoniae*	*bla*CTX-M-3, *bla*SHV, *bla*TEM	*emrD*, *kdeA*, *oqxA10*, *oqxB5*, *silA*	R	R	R	S	S	R	R	R	I	R	S	S	S	R	R	S
B5	*K. pneumoniae*	*bla*CTX-M-15, *bla*SHV	*emrD*, *kdeA*, *oqxA*, *oqxB*, *silA*	R	SDD	R	S	I	R	S	R	R	S	S	S	S	S	S	S
B6	*K. pneumoniae*	*bla*CTX-M-65, *bla*OXA-10, *bla*SHV, *bla*TEM	*emrD*, *kdeA*, *arsB*, *oqxA10*, *oqxB*, *silA*	R	SDD	R	S	S	R	R	I	I	S	S	S	S	R	R	S
B7	*K.pneumoniae*	*bla*CTX-M-3, *bla*SHV, *bla*TEM	*emrD*, *kdeA*, *oqxA*, *oqxB19*, *arsB*, *silA*	R	R	R	S	S	R	R	R	R	S	S	S	S	R	R	S
B8	*H. alvei*	*bla*ACC	N/A	R	S	R	R	R	R	S	S	R	S	S	S	R	S	S	S
B9	*H. alvei*	*bla*ACC	N/A	R	S	R	S	R	R	S	S	R	S	S	S	R	S	S	S
B10	*C. freundii*	*bla*CMY	*arsA*, *arsB*	R	S	R	R	R	R	S	R	R	S	S	S	S	S	S	S
B11	*C. freundii*	*bla*CMY	*arsA*, *arsB*	R	SDD	R	R	R	R	S	R	I	S	I	S	R	S	S	S
B12	*C. braakii*	*bla*CMY, *bla*CTX-M-15	N/A	R	R	R	R	R	R	S	R	R	S	S	S	S	S	S	S
B13	*P. hauseri*	*bla*DHA, *hugA*	N/A	R	S	R	R	R	S	R	R	R	I	I	S	S	R	R	S
B14	*E. cloacae*	*bla*ACT	*oqxA*, *oqxB*	R	S	R	R	R	R	S	R	R	S	S	S	S	S	S	S

AMP: ampicillin; FEP: cefepime; CTX: cefotaxime; FOX: cefoxitin; CAZ: ceftazidime; CRO: ceftriaxone; C: chloramphenicol; CIP: ciprofloxacin; CS: colistin; CN: gentamicin; IPM: imipenem; MEM: meropenem; NA: nalidixic acid; TE: tetracycline; SXT: co-trimoxazole; DOR: doripenem; S: susceptible; I: intermediate; R: resistant; SDD: susceptible-dose dependent.

**Table 3:** Six Chinese medicinal herb extracts that demonstrated broad-spectrum anti-ESBL-PE activities against 14 different ESBL-PE strains, as determined by the disc diffusion tests.

Herb	Extraction	Zone of Inhibition (Mean ± S.D., mm)													
		ESBL-PE Strains													
		B1	B2	B3	B4	B5	B6	B7	B8	B9	B10	B11	B12	B13	B14
*L. erythrorhizon*	W	-	-	-	-	-	11.35 ± 0.37	-	8.12 ± 0.33	8.20 ± 0.15	8.97 ± 0.21	7.29 ± 0.04	8.54 ± 0.47	9.49 ± 0.09	-
	E	-	-	-	-	-	8.50 ± 0.16	-	-	-	6.54 ± 0.25	-	6.93 ± 0.07	7.78 ± 0.16	-
	UAE	-	-	-	-	-	9.69 ± 0.34	-	6.76 ± 0.06	6.80 ± 0.09	7.31 ± 0.34	-	7.73 ± 0.13	8.53 ± 0.14	-
*S. glabra*	W	-	-	-	-	-	10.37 ± 0.18	-	9.33 ± 0.04	8.13 ± 0.59	6.79 ± 0.11	-	7.36 ± 0.09	10.07 ± 0.05	-
	E	-	-	-	-	-	7.79 ± 0.15	-	-	-	-	-	-	-	-
	UAE	-	-	-	-	-	8.43 ± 0.06	-	-	-	-	-	-	7.10 ± 0.09	-
*S. baicalensis*	W	-	-	-	-	-	12.06 ± 0.03	-	9.74 ± 0.42	8.81 ± 0.05	8.85 ± 0.21	-	7.78 ± 0.10	7.73 ± 0.06	-
	E	-	-	-	-	-	10.79 ± 0.06	-	9.48 ± 0.07	8.47 ± 0.23	7.59 ± 0.17	-	8.01 ± 0.15	7.06 ± 0.13	-
	UAE	-	-	-	-	-	11.37 ± 0.24	-	9.68 ± 0.01	8.63 ± 0.11	8.15 ± 0.11	-	8.43 ± 0.03	7.40 ± 0.16	-
*C. chinensis*	W	-	-	-	-	-	12.81 ± 0.22	-	10.37 ± 0.20	10.10 ± 0.17	6.82 ± 0.15	-	-	11.09 ± 0.08	-
	E	9.69 ± 0.55	10.54 ± 0.18	10.68 ± 0.27	-	-	11.73 ± 0.16	-	9.86 ± 0.18	10.29 ± 0.14	-	-	-	10.44 ± 0.21	-
	UAE	11.17 ± 0.44	10.68 ± 0.05	11.46 ± 0.21	-	-	13.38 ± 0.22	-	10.12 ± 0.11	10.48 ± 0.23	6.95 ± 0.10	-	-	10.98 ± 0.18	-
*P. vulgaris*	W	-	-	-	-	-	8.05 ± 0.34	-	-	-	6.67 ± 0.33	-	-	7.07 ± 0.19	-
	E	-	-	-	-	-	9.77 ± 0.10	-	8.14 ± 0.11	-	6.81 ± 0.05	-	6.84 ± 0.09	8.84 ± 0.08	-
	UAE	-	-	-	-	-	10.92 ± 0.08	-	7.95 ± 0.47	-	7.86 ± 0.17	7.22 ± 0.15	7.74 ± 0.32	9.55 ± 0.18	-
*M. officinalis*	W	-	-	-	-	-	11.77 ± 0.03	-	9.07 ± 0.19	7.19 ± 0.17	-	-	7.04 ± 0.01	10.92 ± 0.06	-
	E	-	-	-	-	-	7.75 ± 0.18	-	-	-	-	-	-	7.95 ± 0.14	-
	UAE	-	-	-	-	-	8.81 ± 0.03	-	6.42 ± 0.21	-	-	-	-	8.85 ± 0.04	-

W: water decoction; E: 80% ethanol maceration; UAE: 50% ethanol extraction assisted with ultrasound; B1–B3: *Escherichia coli*; B4–B7: *Klebsiella pneumoniae*; B8–B9: *Hafnia alvei*; B10–B11: *Citrobacter freundii*; B12: *Citrobacter braakii*; B13: *Proteus hauseri;* B14: *Enterobacter cloacae*; -: no inhibitory activity.

**Table 4:** Minimum inhibitory concentrations of six Chinese medicinal herb extracts against various types of ESBL-PE strains.

Herb	Extraction	Minimum Inhibitory Concentration (mg/mL)					
		ESBL-PE Isolates					
		*E. coli* B2	*K. pneumoniae* B6	*H. alvei* B8	*C. freundii* B10	*C. braakii* B12	*P. hauseri* B13
*L. erythrorhizon*	W	12.5	1.5625	3.125	3.125	6.25	1.5625
	E	25	12.5	6.25	12.5	12.5	12.5
	UAE	25	1.5625	3.125	3.125	6.25	3.125
*S. glabra*	W	12.5	3.125	6.25	3.125	6.25	3.125
	E	25	12.5	6.25	12.5	25	12.5
	UAE	25	3.125	6.25	6.25	12.5	3.125
*S. baicalensis*	W	12.5	3.125	6.25	3.125	3.125	3.125
	E	12.5	1.5625	3.125	3.125	3.125	1.5625
	UAE	12.5	1.5625	3.125	3.125	3.125	1.5625
*C. chinensis*	W	3.125	1.5625	3.125	3.125	3.125	3.125
	E	1.5625	6.25	3.125	3.125	1.5625	3.125
	UAE	1.5625	3.125	3.125	3.125	1.5625	3.125
*P. vulgaris*	W	25	3.125	3.125	3.125	3.125	3.125
	E	>25	3.125	6.25	6.25	12.5	3.125
	UAE	25	3.125	6.25	3.125	3.125	3.125
*M. officinalis*	W	25	1.5625	3.125	3.125	3.125	1.5625
	E	25	1.5625	3.125	3.125	3.125	1.5625
	UAE	25	0.78125	3.125	3.125	3.125	1.5625

W: water decoction; E: 80% ethanol maceration; UAE: 50% ethanol extraction assisted with ultrasound.

**Table 5:** The quantitative content of seven alkaloids (%) in *Coptis chinensis* extracts.

Alkaloid Compounds	Extracts of *C. chinensis*		
	W (%)	E (%)	UAE (%)
Berberine	17.9	13.4	23.5
Palmatine	7.6	5.3	9.0
Coptisine	3.7	2.4	5.0
Epiberberine	8.6	5.5	10.9
Jatrorrhizine	2.3	1.6	2.8
Columbamine	1.4	1.1	1.7
Groenlandicine	0.9	0.6	1.2
Total	42.4	29.9	54.1

W: water decoction; E: 80% ethanol maceration; UAE: 50% ethanol extraction assisted with ultrasound. The percentage content of alkaloids in the *C. chinensis* extract was calculated as the percentage of the weight of alkaloids in the *C. chinensis* extract relative to the total weight of the *C. chinensis* extract.

**Table 6:** Minimum inhibitory concentrations of berberine and *Coptis chinensis* UAE extract on the growth of ESBL-PE strains.

ESBL-PE Strains	Berberine (mg/mL)	*C. chinensis* with UAE Extract (mg/mL) *	Equivalent Berberine Concentration in *C. chinensis* with UAE Extract (mg/mL)
*E. coli* B2	0.75	1.5625	0.368
*K. pneumoniae* B6	3.00	3.125	0.735
*H. alvei* B8	0.75	3.125	0.735
*C. freundii* B10	3.00	3.125	0.735
*C. braakii* B12	0.75	1.5625	0.368
*P. hauseri* B13	1.50	3.125	0.735

* *C. chinensis* UAE extract contains 23.5% berberine.

**Table 7:** The twenty Chinese medicinal herbs used in this study.

Scientific Name and Part of Plant Used	Place of Origin	Pharmacology [62]	Phytoconstituents [62]
*Scleromitrion diffusum* (willd) R.J.Wang (Leaves)	Jiangxi	Anti-tumor and anti-inflammatory	Asperuloside, organic acids and their esters, anthraquinones, triterpenes
*Houttuynia cordata* Thunb. (Whole plant)	Guangdong	Anti-viral, antibacterial, immunity-strengthening, etc.	Volatile oil and flavonoids
*Lithospermum erythrorhizon* Siebold & Zucc. (Root)	Xinjiang	Anti-inflammatory, anti-pathogenic microorganism, pain-killing, etc.	Shikonin, acetylshikonin, deoxyshikonin
*Wurfbainia villosa* (Lour.) Škorničk. & A.D.Poulsen (Fruit)	Guangdong	Anti-platelet aggregation, anti-ulcer and pain-relieving	Bornyl acetate, camphor, and limonene
*Scrophularia ningpoensis* Hemsl. (Root)	Hubei	Preserving effects on the cardiovascular system, CNS-inhibiting, etc.	Iridoids compounds, ningpogenin, ningpogosides A and B
*Portulaca oleracea* L. (Whole plant)	Jiangsu	Antibacterial, uterus-contracting, blood-fat-lowering, etc.	Noradrenaline, dopa, dopamine, betanidin, oxalic acid, triterpene
*Grona styracifolia* (Osbeck) H.Ohashi & K.Ohashi. (Whole plant)	Sichuan	Anti-inflammatory, pain-killing, etc	Alkaloid, flavone flycosides, phenols, tannin
*Lonicera japonica* Thunb. (Flower bud)	Henan	Antimicrobial, anti-inflammatory, antifebrile, blood-fat-lowering, etc.	Chlorogenic acid, ginnol, isochlorogenic acid, β-sitosterol, linalool, etc.
*Gardenia jasminoides* J.Ellis (Fruit)	Hubei	Hepatic-protective, anti-inflammatory	Flavonoid genipin, pectin, and tannin
*Atractylodes macrocephala* Koidz. (Rhizoma)	Zhejiang	Gastric-ulcer-preventing, intestines-movement-influencing, anti-bacteria, etc.	Atratylone, eudesmol, palmitic acid, hinesol, humulene, sesquiterpene lactones, polyynealcohols
*Atractylodes lancea* (Thunb.) DC. (Rhizoma)	Zhejiang	Gastric-secretion-restraining, influential on the liver, blood sugar and stomach and intestinal movement, etc.	Volatile oil, atraclyloin, atractylon, chamigrene, caryophyllene, elemene, atractylodin, furaldehyde, tryptophane, etc.
*Sarcandra glabra* (Thunb.) Nakai (Whole plant)	Guangdong	Anti-tumors, antibacterial, antivirotic, etc.	Coumarin, flavonoid glycosides, essential oils, tannin
*Scutellaria baicalensis* Georgi (Root)	Hebei	Antibacterial, anti-inflammatory, heart-brain-protecting, hepatic-protective, immunity-regulating and anti-tumor and antiallergic	Baicalensis and baicalein
*Forsythia suspensa* (Thunb.) Vahl (Fruit)	Hebei	Antimicrobial, anti-inflammatory, etc.	Lignans, flavonoids, benzene ethane derivatives
*Glycyrrhiza uralensis* Fisch. (Root)	Inner Mongolia	Anti-viral, antibacterial, anti-ulcer, atitussive, anti-inflammatory, phlegm-removing, anti-tumor, etc.	Triterpenoid saponins
*Chrysanthemum morifolium* Ramat. (Flower)	Hangzhou	antibacterial and cardiovascular-system-affecting	Borneol, chrysanthenone, camphor, acacetin-7-rhamnoglucoside, indican, thymol, apigenin, luteolin, cosmosiin, volatile oils
*Coptis chinensis* Franch. (Root)	Sichuan	Antimicrobial, antiprotozoal, beneficial to cyclic system and nervous system, anti-arrhythmia, anti-ulcer, beneficial to the gallbladder, anti-tumor and anti-inflammatory	Berberine, epiberberine, coptisine, berberrubine, palmatine, jatrorrhizine, worenine, magnoflorine, ferulic acid, obakunone, obakulactone
*Prunella vulgaris* L. (Fruit spike)	Henan	Anti-hypertension, hyperglycemic, antibacterial, anti-viral, etc.	Saponin, ursolic acid, tartaric acid, prunellin
*Magnolia officinalis* Rehder & E.H.Wilson (Bark)	Sichuan	Anti-ulcerative, anti-pathogenic microorganism and anti-tumors, anti-patelet, etc.	Lignan, monoterpene lignin, norlignan, honokiol, camphor
*Sophora flavescens* Aiton (Root)	Henan	Anti-inflammatory, anti-pathogenic microorganism, etc.	Alkaloids, flavonoids

## Data Availability

The corresponding author will provide the data used in this study upon reasonable request.

## Abbreviations

The following abbreviations are used in this manuscript:

ESBL-PE	Extended-Spectrum β-Lactamase-Producing-*Enterobacterales*
IZD	inhibition zone diameter
MALDI-TOF MS	Matrix-Assisted Laser Desorption Ionization Time-Of-Flight Mass Spectrometry
MIC	minimum inhibitory concentration
UAE	ultrasound-assisted extraction
